# Epidemiological survey of orthopedic joint dislocations based on nationwide insurance data in Taiwan, 2000-2005

**DOI:** 10.1186/1471-2474-12-253

**Published:** 2011-11-05

**Authors:** Nan-Ping Yang, Hou-Chaung Chen, Dinh-Van Phan, I-Liang Yu, Yi-Hui Lee, Chien-Lung Chan, Pesus Chou, Jenn-Huei Renn

**Affiliations:** 1 Community Health Research Center & Institute of Public Health, National Yang-Ming University, Taipei, Taiwan; 2 Department of Orthopedics & Department of Medical Research, Tao-Yuan General Hospital, Department of Health, Taoyuan, Taiwan; 3 Department of Orthopedics, Tai-Chung General Hospital, Department of Health, Taichung, Taiwan; 4 Department of Information Management, Yuan-Ze University, Taoyuan, Taiwan; 5 Department of Nursing, School of Nursing, Chang-Gang University, Taoyuan, Taiwan; 6 Department of Orthopedics, Kaohsiung Veterans General Hospital, Executives Yuan, Kaohsiung, Taiwan

**Keywords:** incidence, orthopedic dislocation

## Abstract

**Background:**

The epidemiology of acute orthopedic dislocations is poorly understood. A nationwide database provides a valuable resource for examining this issue in the Taiwanese population.

**Methods:**

A 6-year retrospective cohort study of 1,000,000 randomly-sampled beneficiaries from the year 2005 was used as the original population. Based on the hospitalized and ambulatory data, the concomitant ICD9-CM diagnosis codes and treatment codes were evaluated and classified into 8 and 3 major categories, respectively. The cases matching both inclusive criteria of dislocation-related diagnosis codes and treatment codes were defined as incident cases.

**Results:**

During 2000-2005, the estimated annual incidence (per 100,000 population) of total orthopedic dislocations in Taiwan was 42.1 (95%CI: 38.1-46.1). The major cause of these orthopedic dislocations was traffic accidents (57.4%), followed by accident falls (27.5%). The annual incidence dislocation by location was shoulder, 15.3; elbow, 7.7; wrist, 3.5; finger, 4.6; hip, 5.2; knee, 1.4; ankle, 2.0; and foot, 2.4. Approximately 16% of shoulder dislocations occurred with other concomitant fractures, compared with 17%, 53%, 16%, 76% and 52%, respectively, of dislocated elbow, wrist, hip, knee, and ankle cases. Including both simple and complex dislocated cases, the mean medical cost was US$612 for treatment of a shoulder dislocation, $504 for the elbow, $1,232 for the wrist, $1,103 for the hip, $1,888 for the knee, and $1,248 for the ankle.

**Conclusions:**

In Taiwan, three-quarters of all orthopedic dislocations were of the upper limbs. The most common complex fracture-dislocation was of the knee, followed by the wrist and the ankle. Those usually needed a treatment combined with open reduction of fractures and resulted in a higher direct medical expenditure.

## Background

Musculoskeletal injuries are a major public health concern globally and contribute a large burden of disability and suffering [1]. In 2001, injuries in developing countries accounted for 11% of the world's disease burden, and ranked 11th of all causes of both mortality and morbidity [2]. The global burden of injury is surprising: injuries are predicted to become a leading cause of death and disability over the next few decades, and decreasing the burden of injuries is among the main challenges in the arena of public health over the coming century [3,4]. As in Taiwan, it is essential for public health officials of all countries to have a good understanding of the magnitude and characteristics of the national extremities trauma or fracture rates in order to develop and evaluate injury prevention programs. In Taiwan, injury or poisoning is the most common diagnostic category (26.4%) in the emergency unit, and the general cost of emergency medical care has been shown to increase with the age of the patient [5]. The prevalence of various definite orthopedic fractures increases with age, and a higher prevalence of orthopedic fractures were found in Taiwan [6]. A nationwide database provides a valuable resource for examining this issue in the Taiwanese population. The aims of the current study were: (1) to reveal the treatment incidences of various orthopedic dislocations, the major cause of these dislocations and their re-dislocation proportion in Taiwan; and (2) to understand the medical burden of these injuries and their related factors, such as injury classifications and treatment methods.

## Methods

### Source, security, and quality control of data

Taiwan launched a single-payer National Health Insurance (NHI) Program on March 1, 1995. As of 2007, 22.60 million of Taiwan's total population of 22.96 million were enrolled in this program; foreigners in Taiwan are also eligible for inclusion. This universal national health insurance, financed jointly by payroll taxes, subsidies, and individual premiums, commenced in Taiwan and its coverage expanded from 57% of the population (before the introduction of national health insurance) to more than 98% (after the year of 2005). All the enrollees enjoy almost free access to healthcare with small co-payment by most clinics and hospitals [7,8]. In order to respond to current and emerging health issues rapidly and effectively, the National Health Research Institute (NHRI), in cooperation with the National Health Insurance Bureau (NHIB), established a nationwide research database. The NHRI safeguards the privacy and confidentiality of those included in the database and routinely transfers health insurance data from the NHIB to enable health researchers to analyze and improve the health of Taiwan's citizens. The NHI database contains registration files and original claims data for reimbursement, and access to the National Health Insurance Research Database (NHIRD), which was derived from this system by the NHIB and is maintained by the NHRI, is provided to scientists in Taiwan for research purposes.

The NHIB has established a uniform system to control the quality of medical services and coding. If the medical services provided to beneficiaries by the contracted medical care institution are determined by the Professional Peer Review Committee to be incompatible with the provisions of the NHI Act, the expenses thereof are borne by the contracted medical care institution themselves. Otherwise, the Disputes Settlement Board, established under the NHI scheme, settles disputes arising in cases approved by the insurer and raised by the insured, group insurance applicants, or contracted medical care institutions.

### Data protection and permission

Data in the NHIRD that could be used to identify patients or care providers, including medical institutions and physicians, is scrambled before being sent to the NHRI for database inclusion, and is further scrambled before being released to each researcher. Theoretically, it is impossible to query the data alone to identify individuals at any level using this database. All researchers who wish to use the NHIRD and its data subsets are required to sign a written agreement declaring that they have no intention of attempting to obtain information that could potentially violate the privacy of patients or care providers. This study protocol was evaluated by the NHRI, who gave their agreement to the planned analysis of the NHIRD (Application and Agreement Number: 98018). This study was also approved by the Institutional Review Board (IRB) of Taoyuan General Hospital, which has been certificated by the Department of Health, Taiwan (IRB Approval Number: TYGH97040). The study was also supported by a grant from the same hospital (Grant Number: DOH-PTH-9817).

### Data selection and definition of orthopedic dislocations

Every claimant of the NHI Program at any time during 2005 was included in the population (22,717,053 people) selected for random sampling. Using a random number generator to produce 1,073,891 random numbers, the original claims data of 1,000,000 randomly-sampled beneficiaries from the year 2005 was the target population of the present study. The majority of the sample population of 1,000,000 subjects were aged from 20 to 65 years, and the male and female subpopulations were of a similar size (49.6% *vs*. 50.4%, respectively).^6 ^There were no significant differences in the gender distribution, age distribution or average insured payroll-related amount between the claimants in the 2005 sampling data and the original NHIRD. As a retrospective cohort study population, the registration and claims data of these 1,000,000 individuals collected by the NHI Program were traced back for the previous 5 years (2000-2004), and two separate categories of expenditure were used as below: (1) inpatient expenditure by admission (DD files); (2) ambulatory care expenditure by visit (CD files). The beneficiaries within both the CD and DD files of the LHID2005 during 2000-2005 formed the target population in this study.

In order to investigate the incidence of orthopaedic dislocations in the hospitalized and ambulatory subjects included in this study, the concomitant ICD9-CM diagnosis codes and treatment codes were evaluated and classified into 8 and 3 major categories, respectively. The cases matching both inclusive criteria of dislocation-associated diagnosis codes and treatment codes were selected. The major diagnosis codes of orthopaedic dislocation categories were defined as coded as the 831 series (shoulder dislocation), 832 series (elbow dislocation), 833 series (wrist dislocation), 834 series (finger dislocation), 835 series (hip dislocation), 836 series (knee dislocation), 837 series (ankle dislocation), and 838 series (foot dislocation); the major orthopaedic dislocation-associated treatment codes were defined as coded as the 79.7 series (closed reduction), 79.8 series (open reduction), and 79.0 series to 79.6 series (open reduction of bony fractures).

### Statistics

Descriptive statistics and annual incidence rates (per 100,000 population) of specific dislocation types and 95% confidence intervals for the estimated rates were calculated based on the number of observed cases and person-years.

## Results

### Basic characteristics of the enrolled subjects in Taiwan, 2000-2005

**Figure 1:**
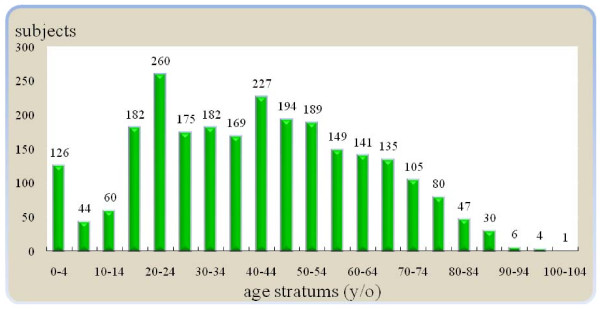
**Age-distributions of the various joints-dislocated subjects in Taiwan, 2000-2005**.

According to the two inclusion criteria of an ICD9_CM coding as an orthopedic dislocation and the receipt of related treatment, 344 people were enrolled in the present study in 2000, and 361, 374, 426, 467, and 534 subjects were enrolled in 2001, 2002, 2003, 2004, and 2005, respectively. According to the E-code (coding for an external cause of injury) of ICD9_CM coding of the available data (1,244 of the 2,506 enrolled subjects), the major mechanisms of these orthopedic dislocations were traffic accidents, followed by accident falls. The majority of these selected people with dislocations were male (62.4%) and middle-aged (20-59 years-old) (Table 1). Especially, the two peak age-stratum of various joints dislocation were occurred at 20-24 years-old and 40-44 years-old (Figure 1).

**Table 1:** Basic Characteristics of Enrolled Subjects, 2000-2005.

	2000	2001	2002	2003	2004	2005
Gender						
male	217	225	211	264	293	354
female	127	136	163	162	174	180
Age Stratum						
< 20-years-old	72	59	45	65	82	89
20-39-years-old	104	119	124	131	154	154
40-59-years-old	100	104	113	135	129	178
≥ 60-years-old	68	79	92	95	102	113
Total	344	361	374	426	467	534
External Causes of Injury*						
transport accidents	124	101	115	103	150	121
accident falls	39	51	51	47	56	98
others	34	29	25	26	32	42

*: according to E-code of ICD9_CM

### The estimated incidence of various orthopedic dislocations in Taiwan, 2000-2005

Table 2 reveals detailed the case distributions of the 8 major categories of orthopedic dislocations during 2000-2005. In general, 348 cases of dislocation occurred in 2000, and 362, 376, 430, 469, and 540 cases occurred in 2001, 2002, 2003, 2004, and 2005, respectively. During 2000-2005, the averaged annual incidence of all orthopedic dislocations in Taiwan was estimated to be 42.1 cases per 100,000 persons, with a 95%CI of 38.1 to 46.1 per 100,000. The averaged annual incidences of different locations of dislocation in Taiwan are shown in Table 2, and ranged from 1.4 (95% CI 0.7-2.1) per 100,000 for knee to 15.3 (95% CI 12.9-17.8) per 100,000 for shoulder.

**Table 2:** Estimated Incidence* of Various Joint Dislocation Cases in Taiwan, 2000-2005.

	2000 (cases No.)	2001 (cases No.)	2002 (cases No.)	2003 (cases No.)	2004 (cases No.)	2005 (cases No.)	Average Incidence*, 2000-2005	(95% Confidence Interval)
Shoulder (831.X)	132	130	127	187	173	170	15.3	(12.9-17.8)
Elbow (832.X)	53	64	70	81	84	107	7.7	(5.9-9.4)
Wrist (833.X)	30	37	30	24	36	52	3.5	(2.3-4.6)
Finger (834.X)	34	43	44	42	46	69	4.6	(3.3-6.0)
Hip (835.X)	48	39	56	39	55	75	5.2	(3.8-6.6)
Knee (836.X)	12	11	15	14	16	16	1.4	(0.7-2.1)
Ankle (837.X)	20	14	17	22	27	21	2.0	(1.1-2.9)
Foot (838.X)	19	24	17	21	32	30	2.4	(1.4-3.3)
Total	348	362	376	430	469	540		
Annual Incidence*	*34.8*	*36.2*	*37.6*	*43.0*	*46.9*	*54.0*	42.1	(38.1-46.1)

*: 1/100,000

### Classifications and treatments of six major dislocations in Taiwan, 2000-2005

Six major orthopedic dislocations, their different injury classifications and treatment methods were analyzed in the present study (Table 3). Approximately 16% of shoulder dislocations occurred with other concomitant fractures, compared with 17%, 53%, 16%, 76% and 52%, respectively, of dislocated elbow, wrist, hip, knee, and ankle cases. The treatment categories were divided into simple closed reduction, simple open reduction, and combined associated-fractures treatment. The method most commonly used to treat a dislocated shoulder, elbow, and hip was closed reduction (the percentages among all treatment methods being 59.7%, 73.0%, and 74.2%, respectively). Nearly all the complicated fracture-dislocations were needed to receive some surgical interventions involving the fracture management. More combined with more complicated fracture-dislocations were found in cases of wrist (52.6%), knee (76.2%) and ankle (52.1%) dislocation injuries, and, among them, 51.6%, 71.1% and 50.4% received some surgical intervention to treat their fractures concurrently by internal fixation methods, respectively.

**Table 3:** Classification and Management of Major Joint Dislocation Cases in Taiwan, 2000-2005.

	Classification of Injuries		Treated Methods		
	
	Simple* (%)	Complex* (%)	CR (%)	OR (%)	CX (%)
Shoulder (831.X)	84.3%	15.7%	59.7%	24.7%	15.7%
Elbow (832.X)	83.4%	16.6%	73.0%	10.3%	16.7%
Wrist (833.X)	47.4%	52.6%	22.1%	26.3%	51.6%
Hip (835.X)	83.7%	16.3%	74.2%	8.2%	17.5%
Knee (836.X)	23.8%	76.2%	17.8%	11.1%	71.1%
Ankle (837.X)	47.9%	52.1%	19.2%	30.4%	50.4%

*Simple injury means pure dislocation; complex injury means the dislocation combined with some fractures.

CR: Closed Reduction of Dislocation (79.7X)

OR: Open Reduction of Dislocation (79.8X)

CX: Combined with Open Reduction of Fractures (79.0X to 79.6X)

### The medical burden and re-dislocation rate of six major dislocations in Taiwan, 2000-2005

The direct medical expenditure (i.e. cost) of the six major dislocations were calculated. Including both simple and complex dislocated cases, the mean medical cost was NT$20,180(US$612) for treatment of a shoulder dislocation, NT$16,616 ($504) for the elbow, NT$40,647 ($1,232) for the wrist, NT$36,398 ($1,103) for the hip, NT$62,294 ($1,888) for the knee, and NT$41,182 ($1,248) for the ankle. Among them, re-dislocations were observed for shoulder, elbow, wrist and hip and their aggregated re-dislocation rates were ranged from 0.48% to 12.65% (Table 4).

**Table 4:** Direct Medical Burden and Subsequent Re-dislocation of Major Joint-dislocations in Taiwan, 2000-2005.

	Total Cases	Averaged Medical expenditure	Total Subjects	Subjects with dislocations twice*	Subjects with dislocations 3 times*	Subjects with dislocations 4 or more times*
	
	**No**.	NT$: Mean(SD)	No.(a)	No.(b), %(b/a)	No.(c), %(c/a)	No.(d), %(d/a)
Shoulder (831.X)	919	20,180(29,990)	884	20, 2.26%	6, 0.68%	1, 0.11%
Elbow (832.X)	459	16,616(30,919)	428	14, 3.27%	0, 0.00%	3, 0.70%
Wrist (833.X)	209	40,647(65,263)	208	1, 0.48%	0, 0.00%	0, 0.00%
Hip (835.X)	312	36,398(51,320)	261	23, 8.81%	5, 1.92%	5, 1.92%
Knee (836.X)	84	62,294(48,660)	84	0, 0.00%	0, 0.00%	0, 0.00%
Ankle (837.X)	121	41,182(45,115)	121	0, 0.00%	0, 0.00%	0, 0.00%

*: The mean durations(mean ± SD, month) between index dislocation and subsequent re-dislocation were below: shoulder(10.55 ± 12.42 ms), elbow(4.22 ± 6.25 ms), wrist(0.10 ms), and hip(5.97 ± 11.01 ms).

Note: the currency conversion rate is US$1 = NT$33.

## Discussion

Orthopedic joint dislocations are not uncommon among injured people treated by medical services providers. Most studies concerning epidemiologic surveys of various orthopedic dislocations have been hospital-based case series, and rarely have studies focused on estimating the nationwide incidence. Therefore, the epidemiology of acute orthopedic dislocations is poorly understood. In the present study, because of insufficient records of ICD9_CM E-code, the cause or mechanism of the dislocated joint injury could be determined for only 50% of the cases. Based on the limited records of external cause coding, the main mechanisms of these orthopedic dislocations were traffic accidents (57.4%) and accident falls (27.5%).

The occurrences of joint dislocations found in the present study were much lower than the incidences of fractures reported in an epidemiologic study of twelve categorical orthopedic fractures in Taiwan [6]. For example, the occurrence of shoulder dislocation was about 1/10 that of definite humeral fracture; the occurrence of hip dislocation was about 1/30 that of definite femoral neck fracture; the occurrence of ankle dislocation was about 1/60 that of definite ankle fracture; and the occurrence of wrist dislocation was about 1/100 that of definite radial or ulnar fracture. A study to investigate non-work-related traumatic fractures and dislocations referred for orthopedic services in a large population also revealed a higher rate of fractures compared with dislocations (3,440 fractures and 422 dislocations referred for orthopedic services during the three-year study period). The incidence rate of dislocations referred for orthopedic services was 1.04 per 1000 member-years and, members between the ages of fifteen and nineteen years had the highest rate of dislocations referred for orthopedic services [9].

A study of 10 years of United State's records showed that, of all traumatic shoulder dislocations, the overall adjusted-for-ages incidence rate for traumatic shoulder dislocations was at least 11.2/100,000 person-years and the incidence rates were significantly greater for men than for women [10]. Another study based on data of the National Electronic Injury Surveillance System revealed that a total of 8,940 shoulder dislocations were identified, resulting in an overall incidence rate in the United States of 23.9 (95%CI, 20.8 to 27.0) per 100,000 person-years, young age and male sex were noted as risk factors for shoulder dislocation [11]. In summary, shoulder dislocation accounts for nearly 50 percent of all major joint dislocations [12]. The present study showed that for Taiwanese, shoulder dislocation accounted for merely 36.4% of all orthopedic dislocations and 43.7% of six major orthopedic dislocations. Moreover, the unadjusted total incidence of shoulder dislocation in Taiwan was lower at about two-thirds of the nationwide incidence in the US.

Injuries to the elbow, forearm, and wrist account for more than 25 percent of all sports-related injuries [13]. In the present study, 74% of the orthopedic joint dislocations were of the upper limbs, and among them, about half were of the elbow, wrist and finger. The elbow dislocation incidence rate in Taiwan was only 50% of the incidence rate of shoulder dislocation; 73% of those with an elbow dislocation underwent simple closed reduction treatment, but a higher recurrence rate than that of shoulder dislocation was noted. Of particular note, up to 52% of wrist dislocations were associated with fractures that needed to be treated with more advanced methods. An increasing number of patients are undergoing hip arthroplasty, and postoperative dislocation of the prosthesis is a common complication that occurs in 1 to 3% of patients with primary total hip arthroplasty (THA) and in 5 to 20% of patients with a revised THA [14]. In the present study, the hip was the second most common site of dislocation but the most common site of recurrent dislocation with a recurrence incidence of 12.7%. A recent article reviewing epidemiological studies on sports injury, including 227 studies from 38 countries, found that in sports injuries throughout the countries studied, the ankle was the second most commonly injured body site after the knee, and ankle sprain was the most common type of ankle injury [15]. Another epidemiological survey of ankle sprains, based on the National Electronic Injury Surveillance System in the United States, showed an incidence rate of 2.15 per 1000 person-years and the peak incidence of these ankle sprains occurred between fifteen and nineteen years of age [16]. In the present study, knee dislocation was the least common injury of all orthopedic dislocations, and fortunately, no recurrent cases occurred during the study period in Taiwan. Although the fewest dislocations occurred at the site of the knee, the highest percentage of combined associated-fractures treatment was observed for this site. In Taiwan, the annual incidence of ankle or foot dislocations was estimated to be 4.4/100,000.

The burden of surgical disease, including traumatic musculoskeletal conditions, has not previously been addressed in detail. The burden of injuries must be accurately quantified in the same metric that permits comparison with competing health priorities [17]. For example, the study based on hospital discharge registers of 10 European countries revealed large international differences in injury incidence and associated costs related to hospital admissions, with relatively high costs per capita for Austria, followed by Denmark and Norway, and relatively low costs for Spain, England, and Netherlands [18]. In the present study, some complex dislocated injuries (a case of joint dislocation combined with some bony fracture) were noted and the most common was of the knee (76.2%), followed by the wrist (52.6%) and the ankle (52.1%). These complex injuries usually needed a treatment combined with open reduction of the simultaneous fracture and therefore more combined managements were experienced at the above three major dislocations (71.1%, 51.6% and 50.4%, respectively). Furthermore, comparing the direct medical costs of treatments for the different located joints (despite of simple or complex types), the above three principal dislocations still produced more direct medical burden (the mean medical expenditures were US$1,887.7, US$1,231.7 and US$1,247.9, respectively).

There are some limitations of the current study. The present study is a large-sample nationwide survey that could well represent a definitive snapshot of orthopedic injury problems in Taiwan. However, the enrolled subjects' prior instability status, their detailed dislocation types (for example, a pure hip dislocation or a hip dislocation after the arthroplasty or hemiarthroplasty) and spontaneous or formal manual reductions could not be clarified from the NHI data, which were important factors affecting the optimal or successful treatment methods and the possibility of subsequent recurrence.

## Conclusion

In Taiwan, the annual treatment incidence of all orthopedic dislocations was 42.1/100,000, and three-quarters of these dislocations were of the upper limbs. The major mechanism of approximately 50% of included orthopedic dislocations was traffic accidents, followed by accident falls and the most common recurrent dislocation was of the hip, followed by the elbow. The most common complex fracture-dislocation was of the knee, followed by the wrist and the ankle, those usually needed a treatment combined with open reduction of fractures and resulted in a higher direct medical expenditure.

## Competing interests

This study was funded only by Taoyuan General Hospital, Department of Health, Executive Yuan, Taiwan. All authors declare that they have no other conflicts of interest including directorships, stock holding or contracts.

## Authors' contributions

The study was designed by NPY and HCC, and data were gathered and analyzed by DVP, ILY, YHL and CLC. The initial draft of the manuscript was written by NPY, and the accuracy of the data and analyses was ensured by PC and JHR. All authors participated in the preparation of the manuscript and approved the final version.

## Pre-publication history

The pre-publication history for this paper can be accessed here:

http://www.biomedcentral.com/1471-2474/12/253/prepub
